# Thermal requirements, fertility life table and biological parameters of *Cleruchoides noackae* (Hymenoptera: Mymaridae) at different temperatures

**DOI:** 10.7717/peerj.14911

**Published:** 2023-03-13

**Authors:** Luciane Katarine Becchi, Leonardo Rodrigues Barbosa, José Eduardo Serrão, José Cola Zanuncio, Marcus Vinicius Sampaio, Maurício Magalhães Domingues, Carlos Frederico Wilcken

**Affiliations:** 1 Departamento de Proteção Vegetal, Faculdade de Ciências Agronômicas (FCA), Universidade Estadual Paulista, Botucatu, SP, Brazil; 2 Embrapa Florestas, Empresa Brasileira de Pesquisa Agropecuária, Colombo, Paraná, Brazil; 3 Departamento de Biologia Geral, Universidade Federal de Viçosa, Viçosa, MG, Brazil; 4 Departamento de Entomologia/BIOAGRO, Universidade Federal de Viçosa, Viçosa, MG, Brazil; 5 Instituto de Ciências Agrárias, Universidade Federal de Uberlândia, Uberlândia, MG, Brazil

**Keywords:** Biological control, Bronze bug, Eucalyptus spp., Degree-Days, Development, Parasitism

## Abstract

*Cleruchoides noackae* Lin & Huber (Hymenoptera: Mymaridae) was imported to Brazil in 2012, to manage the exotic pest *Thaumastocoris peregrinus* Carpintero & Dellapé (Hemiptera: Thaumastocoridae), which has been damaging eucalyptus plantations. Knowledge of the thermal requirements and the fertility life table of *C. noackae* is important to improve mass rearing methods for this parasitoid and the effectiveness of its release to manage *T. peregrinus*. The objective was to evaluate the development period, thermal requirements and the fertility life table of *C. noackae* at different temperatures. The egg-adult period of this parasitoid varied from 43 to 14 days at 15 °C and 30 °C, respectively. The emergence of *C. noackae* adults was higher at 15 °C, 18 °C, 21 °C and 24 °C than at 30 °C. Female and male *C. noackae* need 226.75 and 230.41 degree-days and temperatures higher than 10.06 °C and 9.90 °C, respectively, to complete egg-adult development. The number of parasitized eggs per *C. noackae* female was higher at 21 °C, 24 °C and 27 °C, with 5.82, 7.73 and 5.50 eggs, respectively, than at 30 °C (0.45). *Cleruchoides noackae* longevity was greater at 15 °C, 21 °C and 24 °C. The net reproductive rate of the parasitoid was higher at 21 °C and 24 °C than at 30 °C, 3.05, 4.70 and 0.16, respectively. The finite rate of increase of *C. noackae* was greater at 21 °C, 24 °C and 27 °C, than at 30 °C and the intrinsic rate of increase was negative at 30 °C, −0.100. The temperatures 21 °C and 24 °C and from 18 °C to 27 °C are the most adequate for the reproduction and population increase of *C. noackae* parasitizing eggs of *T. peregrinus*, respectively.

## Introduction

The Brazilian climate favors forest plantations with economic and environmental importance for this country (Silva, Costa Sccott & Coronel, 2019), with eucalypts species covering 7.47 million ha (Indústria Brasileira de Árvores (IBÁ), 2021). Exotic pests in Brazilian forest plantations, mainly from Australia, including the bronze bug *Thaumastocoris peregrinus* Carpintero & Dellapé (Hemiptera: Thaumastocoridae) (Carpintero & Dellapé, 2006; Wilcken et al., 2010) have reduced eucalypt yield (Jacobs & Nesser, 2005; Noack & Coviella, 2006; Soliman et al., 2012). The sap-sucking feeding habit of *T. peregrinus* decreases the photosynthesis rate, causing drying and defoliation (Jacobs & Nesser, 2005; Button, 2007; Wilcken et al., 2010).

The egg parasitoid *Cleruchoides noackae* Lin & Huber, 2007 (Hymenoptera: Mymaridae) is the main agent for the biological control of *T. peregrinus* (Lin, Huber & Salle, 2007; Nadel & Noack, 2012). This natural enemy parasitizes *T. peregrinus* eggs in Australia (Lin, Huber & Salle, 2007) and imported to Brazil in 2012 to manage this pest (Wilcken et al., 2014). The emergence rate and sex ratio of this parasitoid in the laboratory and in the field in Minas Gerais state, Brazil was 53% and 52% (Barbosa et al., 2017a) and 0.76 and 0.65, respectively (Barbosa et al., 2018, 2019). *Cleruchoides noackae* adult longevity varied from 2 to 4 days without or with food, respectively (Souza et al., 2016).

Insects are ectothermic (Denis et al., 2011) and temperature changes accelerate or reduce their metabolic processes, behavior and physiology (Gillooly et al., 2002; Abram et al., 2017), emergence rate (Pilkington & Hoddle, 2006; Valente et al., 2017), longevity (Keil, Cummings & de Magalhães, 2015; Souza et al., 2016), survival and parasitism (Hance et al., 2007; Colinet, Boivin & Hance, 2007; Bueno, Parra & de Freitas Bueno, 2012), sex ratio (Moiroux, Brodeur & Boivin, 2014), foraging (Denis et al., 2011) and development (Damos & Savopoulou-Soultani, 2012; Bari, Jahan & Islam, 2015; Laumann & Sampaio, 2020).

The fertility life table and thermal requirements are important to assess the potential for establishment in the field, to compare the life cycle and fertility between *T. peregrinus* and *C. noackae*, to predict the duration and number of generations under different environmental conditions and to define the methods for mass rearing this parasitoid (Pratissoli et al., 2004; Gullan & Cranston, 2017). However, this information is not known for *C. noackae*.

Population growth of insects can be estimated by indexes of the fertility life tables (Haddad, Parra & Moraes, 1999; Maia, Luiz & Campanhola, 2000). Tests with controlled temperatures are useful to determine the biological parameters and thermal requirements of insects (Laumann & Sampaio, 2020). The optimal temperature for development varies with insect species (Lactin et al., 1995; Briere et al., 1999). The fertility life table shows the net reproductive rate (R_o_) (population increase per generation), net rate of population increase (r_m_) (innate capacity of the population increase), finite rate of increase (λ) (number of females added to the population per female per day) and the interval between the generations of the insect (T) (period between the end of the current generation and the next one) (Birch, 1948; Fragoso et al., 2019). These data allow researchers to analyze the population dynamics of insects under different environmental conditions and the potential of the natural enemies for pest control (Van Lenteren, 2009; Laumann & Sampaio, 2020).

The use of *C. noackae* as a biocontrol agent for *T. peregrinus* has shown success, but knowledge of the thermal requirements and the fertility life table of this parasitoid are important to complement its mass rearing tactics and the effectiveness of its release to manage *T. peregrinus*. The objective of this study was to determine the development period (egg-adult), the thermal requirements, biological parameters, and fertility life table of *C. noackae* at different temperatures.

## Materials and Methods

### Study site

The study was carried out in the Entomology Laboratory of the Brazilian Agricultural Research Corporation—Embrapa Florestas in Colombo, Paraná, Brazil.

### Host *Thaumastocoris peregrinus*

*Thaumastocoris peregrinus* eggs were obtained from the rearing facility of Embrapa Florestas entomology laboratory. The insects were kept on *Eucalyptus benthamii* Maiden & Cambage (Myrtaceae) branches from three-year-old plants, in 500 mL Erlenmeyer flasks with water and kept in rectangular plastic cages (40 cm long × 35 cm wide × 8 cm high). Every 2 days, new branches were placed near the old ones to facilitate insect migration and feeding. Strips of paper towel (1.5 cm wide × 15.0 cm long) were placed on the leaves of the eucalyptus branches for 24 h as an oviposition site. *Thaumastocoris peregrinus* was kept at 24 ± 2 °C, 60 ± 10% relative humidity, and 12:12 h (light: dark) photoperiod (Barbosa et al., 2016).

### Parasitoid *Cleruchoides noackae*

The parasitoid *C. noackae* was obtained from the rearing facility of the Embrapa Florestas entomology laboratory. Adult parasitoids were kept in transparent polystyrene flasks (7.5 cm high × 3.0 cm in diameter) with strips of filter paper (7.0 cm high × 1.5 cm wide) moistened with 50% aqueous honey solution as food. *Cleruchoides noackae* was multiplied in *T. peregrinus* eggs, from 0 to 24 h old at 24 ± 2 °C, of 60 ± 10% relative humidity, and 12:12 h (light: dark) photoperiod (Barbosa et al., 2017b).

### Development (egg-adult) and thermal requirements

Ten 0 to 24 h old *T. peregrinus* eggs were exposed to one mated *C. noackae* female in cages. After 24 h, the females were removed and the *T. peregrinus* eggs kept at a constant temperature of 15 °C, 18 °C, 21 °C, 24 °C, 27 °C or 30 °C, reflecting a range of temperatures the parasitoid is likely to encounter in the field in Brazil, at 60 ± 10% relative humidity, and 12:12 h (light: dark) photoperiod until parasitoid emergence. The emerged parasitoids were counted daily and sexed according to their antennae (Lin, Huber & Salle, 2007). Unhatched *T. peregrinus* eggs were dissected to determine the number of unhatched nymphs, unviable eggs (without emergence or presence of *C. noackae* or *T. peregrinus* embryo) and non-emerged parasitoids (developed adults that did not emerge). The duration of the egg-adult period for *C. noackae* females and males and the percentage of viable eggs were calculated.

The thermal requirements were calculated according to the *C. noackae* developmental duration (egg-adult) using the linear regression equation Y = a + bX, where Y is the inverse of the development (days) and X is the temperature (°C). The lower base temperature (Tb) was estimated by replacing the development inverse with zero, while the thermal constant (K) was determined using the inverse of the linear coefficient (K = 1/b) (Haddad, Parra & Moraes, 1999).

The experimental design was completely randomized, with six treatments (temperatures) and 22 replications, each with 10 *T. peregrinus* eggs per *C. noackae* female.

### Parasitism and longevity

*Cleruchoides noackae* couples, newly emerged at 15 °C (*n* = 32), 18 °C (*n* = 26), 21 °C (*n* = 23), 24 °C (*n* = 26), 27 °C (*n* = 22), and 30 °C (*n* = 20) were individualized in transparent polystyrene flasks (7.5 cm high × 3.0 cm in diameter) and fed a 50% aqueous honey solution in filter article (7.0 cm high × 1.5 cm wide). The experimental design was completely randomized, with six treatments of 15 °C, 18 °C, 21 °C, 24 °C, 27 °C and 30 °C, and 32, 26, 23, 26, 22 and 20 replications, respectively, each with a *C. noackae* couple. Ten *T. peregrinus* eggs (<24 h old) were exposed to parasitism by one *C. noackae* female, daily, at temperatures of 15 °C, 18 °C, 21 °C, 24 °C, 27 °C or 30 °C until their death. The temperature at which the parasitoids were placed on the eggs was the same at which they developed. These eggs were removed daily and stored in transparent polystyrene flasks (7.5 cm long × 3.0 cm in diameter) at 24 ± 2 °C, 60 ± 10% RH and 12:12 h (light: dark) photoperiod, until hatching. The number of *T. peregrinus* nymphs and emerged *C. noackae* adults were counted and sexed. The *T. peregrinus* eggs were dissected, after the complete emergence of this insect, to determine the number of its nymphs and *C. noackae* adults that did not emerge and the number of unviable eggs. The daily and total numbers of parasitized eggs, sex ratio, survival and longevity of *C. noackae* males and females were determined. The number of parasitized eggs was calculated using the equation P = number of emerged + non-emerged parasitoids.

### Statistical analysis

Data of egg-adult *C. noackae* development as a function of time and temperature were submitted to a generalized linear model (GLM) with Gaussian distribution (identity link function) and assessed with the *hnp* function of the R package *hnp* (Demétrio, Hinde & Moral, 2014) and those from females and males compared by the Mann-Whitney U test. Data of percentage of emerged or non-emerged parasitoids, daily and total number of emerged parasitoids from eggs parasitized and *C. noackae* sex ratio were submitted to the generalized linear model (GLM) with binomial distribution (logit link function) (Hinde & Demétrio, 1998). Differences between treatments were evaluated using the multiple comparison Tukey test with *glht* function of the multcomp package (Hothorn, Bretz & Westfall, 2008). The sex ratio was evaluated using the Kruskal-Wallis test. The longevity and survival curve of this parasitoid were estimated using Kaplan-Meier and the means compared by the Log-Rank test. The parameters of the *C. noackae* fertility life table were estimated using interactive methods with the lifetable R package (Maia, Luiz & Campanhola, 2000). The pseudo-values for each parameter of the fertility life table were measured by the Jackknife technique to obtain estimates of uncertainty measures, such as variance, and the averages by temperature were submitted to regression analysis. Statistical analyses were performed using the computer program R, version 3.3.2 (R Core Team, 2017).

## Results

### Development (egg-adult) and thermal requirements

The *C. noackae* development period was longer between 15 °C and 24 °C, 43.94 to 15.59 days for females (F_5.101_ = 3,911.8; *p* < 0.0001) and 43.07 to 15.29 days for males (F_5.95_ = 5,760.20; *p* < 0.0001), respectively, and decreased as temperature increased, stabilizing between 27 °C and 30 °C (Table 1). The development period, for *C. noackae* males and females, was similar at each temperature (Mann-Whitney U > 86.000, d.f. = 1.20, *p* > 0.05, Table 1).

**Table 1:** Development periods from egg to adult of females and males of the parasitoid *Cleruchoides noackae* (Hymenoptera: Mymaridae) and emerged or non-emerged adults (mean ± SE) (%) from *Thaumastocoris peregrinus* eggs (Hemiptera: Thaumastocoridae) at different temperatures (Temp. (°C)) (RH: 60 ± 10% and 12-h photophase).

Temp. (°C)	Development period (days)^(a,b)^		Parasitoid (%)^(a)^	
	Females	Males	Emerged	Non-emerged
15	43.94 ± 0.29Aa	43.07 ± 0.18Aa	49.09 ± 6.09a	8.18 ± 4.08ab
18	28.08 ± 0.17Ab	27.69 ± 0.16Ab	53.63 ± 4.99a	1.36 ± 0.74b
21	22.71 ± 0.11Ac	22.54 ± 0.11Ac	50.00 ± 6.31a	0.45 ± 0.45b
24	15.59 ± 0.11Ad	15.29 ± 0.06Ad	49.09 ± 7.29a	2.27 ± 1.12b
27	14.52 ± 0.10Ae	14.63 ± 0.09Ae	43.18 ± 6.22ab	5.45 ± 1.57ab
30	14.38 ± 0.07Ae	14.32 ± 0.07Ae	35.90 ± 4.99b	12.27 ± 3.99a

**Note:**

Averages followed by the same small letter per column^(a)^ or capital letter per line^(b)^ do not differ by the Tukey test^(a)^ and Mann-Whitney U^(b)^ (*p* < 0.05), respectively.

The emergence of *C. noackae* adults was higher at 15 °C, 18 °C, 21 °C and 24 °C than at 30 °C (F_5,110_ = 6.1819; *p* = 0.0001) (Table 1). Rates of *C. noackae* retained in *T. peregrinus* eggs, that is, those that did not emerge, were higher at 30 °C (12.27%) than at 18 °C, 21 °C and 24 °C (1.36%, 0.45%, 2.27%, respectively) (F_5.10_ = 6.1819; *p* < 0.0001) (Table 1).

**Figure 1:**
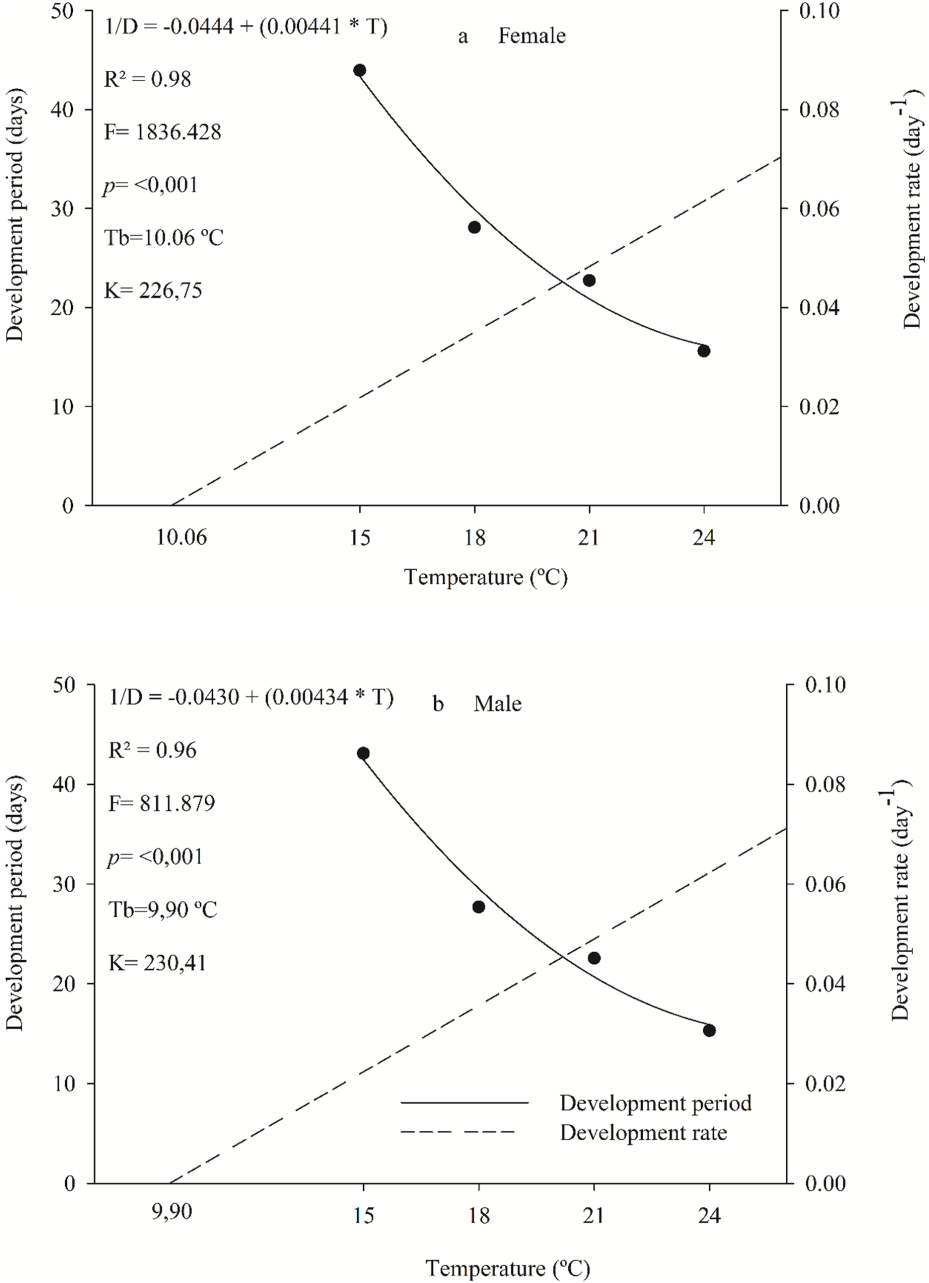
Period and development rate from egg to adult of females (A) and males (B) *Cleruchoides noackae* in eggs of *Thaumastocoris peregrinus* at different temperatures.

Females and males of the parasitoid *C. noackae* completed their development, from egg to adult with 226.75 and 230.41 degree-days with a lower threshold temperature of 10.06 °C and 9.90 °C, respectively (Fig. 1).

### Parasitism and longevity

The number of *T. peregrinus* eggs parasitized daily by *C. noackae* was higher in the first 24 h at 21 °C (F_3.45_ = 8.4864; *p* = 0.0001), 24 °C (F_3.51_ = 36.749; *p* = 0.0001) and 27 °C (F_1.24_ = 9.229; *p* = 0.0058), 4.82; 6.76 and 5.40 eggs, respectively, than at 15 °C (F_4.54_ = 1.0986; *p* = 0.3667), 18 °C (F_2.32_ = 2.96; *p* = 0.0659) and 30 °C (F_2.21_ = 0.9703; *p* = 0.3953) (Table 2).

**Table 2:** Number of eggs of *Thaumastocoris peregrinus* (Hemiptera: Thaumastocoridae) parasitized daily per *Cleruchoides noackae* (Hymenoptera: Mymaridae) female (mean ± SE), at different temperatures (Temp. °C) with relative humidity of 60 ± 10% and 12-h photophase.

		Time (h)				
Temp. (°C)	Parasitized eggs	24	48	72	96	120
15	2.90 ± 0.69C	1.93 ± 0.51CDa	1.68 ± 0.51Aa	0.50 ± 0.18Aa	0.00	0.00
18	3.42 ± 0.74BC	3.19 ± 0.69BCa	0.75 ± 0.20Aa	0.00	–	–
21	5.82 ± 0.83AB	4.82 ± 0.74ABa	1.17 ± 0.50Ab	0.00	1.50 ± 0.44b	–
24	7.73 ± 0.44A	6.76 ± 0.43Aa	1.08 ± 0.30Ab	0.00	0.00	–
27	5.50 ± 0.75AB	5.40 ± 0.72ABa	0.50 ± 0.21Ab	–	–	–
30	0.45 ± 0.19D	0.40 ± 0.18Da	0.00	1.00 ± 0.01Aa	–	–

**Notes:**

– Females did not survive.

Averages followed by the same capital letter, per column, and lowercase letter, per line, do not differ by Tukey’s test (*p* ≤ 0.05).

The total number of *T. peregrinus* eggs parasitized per *C. noackae* female was higher at 21 °C, 24 °C and 27 °C, 5.82, 7.73 and 5.50, respectively than at 30 °C, 0.45 (F_5,143_ = 13.72; *p* < 0.0001) (Table 3).

**Table 3:** Sex ratio and longevity of females and males (mean ± SE) of *Cleruchoides noackae* (Hymenoptera: Mymaridae) at different temperatures (T °C) (RH of 60 ± 10% and 12-h photophase).

T (°C)	Sex ratio^(a)^	Longevity (h)^(b)^	
		Females	Males
15	0.64 ± 0.05a	44.25 ± 4.45a	49.50 ± 5.27a
18	0.67 ± 0.04a	32.30 ± 2.64b	33.23 ± 2.33b
21	0.66 ± 0.05a	51.13 ± 4.60a	42.78 ± 4.51a
24	0.69 ± 0.03a	50.76 ± 3.34a	51.69 ± 4.14a
27	0.57 ± 0.05a	28.36 ± 2.01b	37.09 ± 3.43b
30	0.61 ± 0.07a	28.80 ± 2.80b	28.80 ± 2.20c

**Note:**

Averages followed by the same small letter per column do not differ by the Kruskal-Wallis test^(a)^ and Log-Rank^(b)^ (*p* ≤ 0.05).

The sex ratio of *C. noackae* ranged from 0.57 to 0.69 (*p* = 0.129), with a greater number of females than males emerged between 15 °C and 30 °C (sex ratio >0.5) (Table 3).

The longevity of females (F_5,132_ = 38,481; *p* < 0.0001) and males (F_5,132_ = 24,841; *p* < 0.0001) of *C. noackae* was greater at 15 °C, 21 °C and 24 °C than at 18 °C, 27 °C and 30 °C (Table 3).

**Figure 2:**
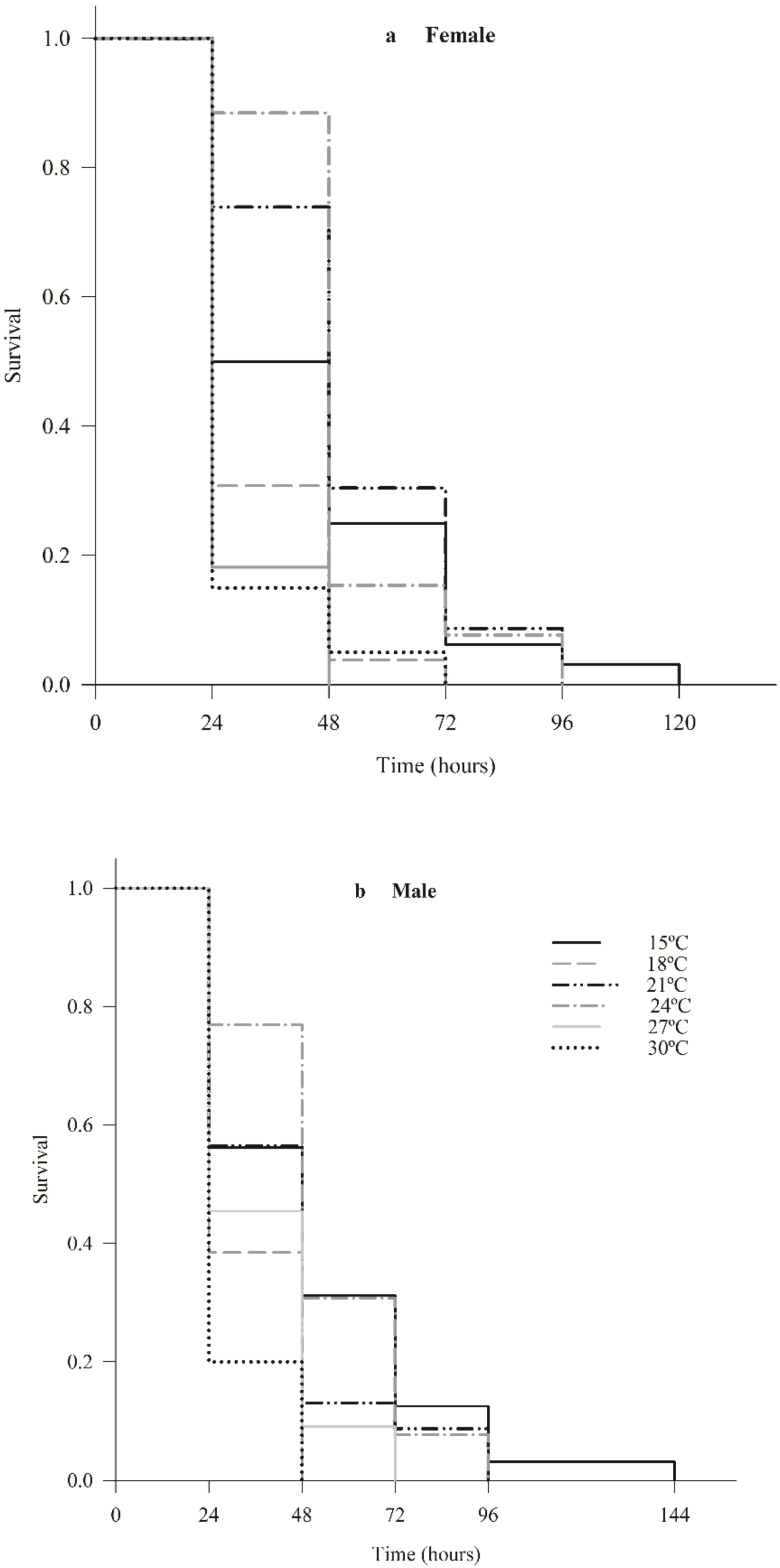
Survival of females (A) and males (B) *Cleruchoides noackae* (Hymenoptera: Mymaridae) at different temperatures.

The survival of female (χ^2^ = 36.7; df = 5; *p* < 0.0001) and male (χ^2^ = 24.7; df = 5; *p* < 0.0001) *C. noackae* was higher than 50% at 15 °C, 21 °C and 24 °C and lower than 20% at 27 °C and 30 °C up to 48 h (Fig. 2).

### Fertility life-table of *Cleruchoides noackae*

The net reproductive rate (R_o_) of *C. noackae* varied from 0.16 to 4.70 between temperatures, with higher values at 21 °C and 24 °C, 3.05 and 4.70, respectively, and lower (0.16) at 30 °C (F_5,143_ = 9.133; *p* < 0.0001). The generation interval (T) of *C. noackae* was longer at 15 °C, 18 °C and 21 °C than at 24 °C, 27 °C and 30 °C (F_5,143_ = 2,190.7; *p* < 0.0001). The finite rate of increase (λ) of *C. noackae* was lower at 30 °C, 0.903 females/female/day, than at 21 °C, 24 °C and 27 °C, 1.050; 1.104 and 1.063, respectively (F_5.143_ = 13.969; *p* < 0.0001). The intrinsic rate of increase (r_m_) of *C. noackae* was higher at 18 °C, 21 °C, 24 °C and 27 °C than at 30 °C, in which it was negative (F_5.143_ = 11.227; *p* < 0.0001) (Table 4).

**Table 4:** Fertility life table of *Cleruchoides noackae* (Hymenoptera: Mymaridae) at different temperatures (Temp. (°C)) with relative humidity of 60 ± 10% and photophase of 12 h).

Temp. (°C)	Fertility life table parameters*			
	R_o_	T	λ	r_m_
15	1.98 ± 0.41bc	43.22 ± 0.22a	1.016 ± 0.01b	0.016 ± 0.01b
18	2.11 ± 0.46bc	28.41 ± 0.24b	1.027 ± 0.01b	0.027 ± 0.01ab
21	3.05 ± 0.49ab	23.02 ± 0.26c	1.050 ± 0.01ab	0.049 ± 0.01ab
24	4.70 ± 0.46a	15.66 ± 0.24d	1.104 ± 0.11a	0.099 ± 0.01a
27	2.53 ± 0.50b	15.45 ± 0.26d	1.063 ± 0.01ab	0.061 ± 0.02ab
30	0.16 ± 0.52c	15.24 ± 0.28d	0.903 ± 0.01c	−0.100 ± 0.02c

**Notes:**

* Net reproductive rate (R_o_), interval between generations (T) (days), finite rate of increase (λ) and intrinsic rate of increase (r_m_).

Averages followed by the same small letter per column do not differ by the Tukey test (*p* ≤ 0.05).

## Discussion

### Development (egg-adult) and thermal requirements

The linear increase in the development period from 15 °C to 24 °C, and the stabilization of this value, for *C. noackae* females and males, between 27 °C and 30 °C may be due to changes in the insect metabolism processes and, consequently, in its development (Abram et al., 2017). This is important because the shorter development reduces the period of exposure of the parasitoid to biotic and abiotic mortality factors and increases its rate of population increase (Laumann & Sampaio, 2020). Similar findings were reported for *C. noackae* in Uruguay, with an egg-adult development period of 27, 24 and 19 days at 18 °C, 20 °C and 22 °C, respectively, (Martínez, González & Dicke, 2018), in South Africa with 15.7 days at 24 °C (Mutitu et al., 2013) and in Brazil with 15 to 16 days at 24 °C (Barbosa et al., 2018). The stabilized development period of *C. noackae* between 27 °C and 30 °C, with 80% mortality after 24 h in this last temperature, may be due to this parameter being near to the upper threshold where the insect does not develop or survive due to lethal action of high temperatures (Lactin et al., 1995; Briere et al., 1999; Damos & Savopoulou-Soultani, 2012). This is similar to that of other Mymaridae with egg-adult development period with a linear pattern between 15 °C to 30 °C of *Anaphes nitens* Girault (Hymenoptera: Mymaridae) in the eggs of the exotic eucalyptus pest *Gonipterus platensis* Marelli (Coleoptera: Curculionidae) (Valente et al., 2017) and *Gonatocerus ashmeadi* Girault (Hymenoptera: Mymaridae) in eggs of *Homalodisca coagulata* Say (Hemiptera: Cicadellidae) (Chen et al., 2006).

The development and emergence of *C. noackae* males and females at the same time and the fact that *T. peregrinus* lays its eggs grouped on eucalyptus leaves (Jacobs & Nesser, 2005) increase the mating possibility of this parasitoid reducing its arrhenotokous parthenogenesis (Mutitu et al., 2013; Becchi et al., 2020), affecting the lifetime reproductive success with increasing proportion of females.

The greater *C. noackae* emergence at 15 °C, 18 °C, 21 °C and 24 °C than at 30 °C may be related to the production of a series of proteins in response to high temperatures that increase the organism tolerance to additional stresses (Hallman & Denlinger, 1998). Moreover, the shortest emergence period at 30 °C may be due to the quality of the *T. peregrinus* host eggs, whose mortality, after 5 days at 35 °C, was 100% (Nadel et al., 2015). Limitations on egg to adult development for *Anaphes inexpectatus* Huber and Prinsloo (Hymenoptera: Mymaridae) and *A. nitens* in *G. platensis* eggs have been reported at 30 °C, with emergence reduced, for each species, to 7% and 0%, respectively (Valente et al., 2017). The reduced developmental success of *C. noackae* at 15 °C (only 8.18% emergence) may be related to constant exposure to this temperature affecting its longevity, fertility and mobility (Turnock & Fields, 2005; Hance et al., 2007), and indicates that on short-term exposures to this temperature, parasitoids can tolerate or recover more easily. *Cleruchoides noackae* emergence from *T. peregrinus* eggs at 21 °C differed from that of this parasitoid for several generations in the laboratory in Uruguay, 20% to 30% at 22 °C (Martínez, González & Dicke, 2018). The parasitoid: host ratio is a defining factor that affects progeny production and sex ratio of parasitoids (Godfray, 1994; Riddick, 2003; Aung et al., 2011). Thus, an accurate understanding of how parasitoid females adjust the number of progenies by host density helps to improve the mass-production of parasitoids (Wajnberg, 2010). Parasitoid: host ratio of 1:10 is the most appropriate for *C. noackae* rearing; therefore, the 1:20 ratio used in Uruguay (Martínez, González & Dicke, 2018) may explain these differences in this research.

The egg-adult development of female and male *C. noackae* parasitoids with 226.75 and 230.41 degree-days (K) and a lower threshold temperature (Tb) of 10.06 °C and 9.9 °C, respectively, indicate the high adaptation of this natural enemy to regions with these climate conditions (Messenger & Flitters, 1958; Damos & Savopoulou-Soultani, 2012). Larger Tb is common for species from tropical regions (Damos & Savopoulou-Soultani, 2012). This is related to the temperatures where this insect is normally found in Brazil, with an average annual value between 24 °C and 25 °C, minimum of 20 °C and maximum of 31 °C (Ramos et al., 2020). In addition, *C. noackae* thermal development requirements match environmental conditions favorable to its host *T. peregrinus*, above 9.93 °C from nymph to adult (Barbosa et al., 2019). This could increase the parasitoid’s efficiency for biological control, and reveals the evolutionary adaptation of parasitoids that favors their success in mass releasing (Van Lenteren, 2009; Laumann & Sampaio, 2020). The thermal constant (K), of *C. noackae* males and females was lower than that for *T. peregrinus*, 338.5 degrees-day (Barbosa et al., 2019), indicating that this parasitoid spends less energy to complete its development and, therefore, has more generations per period than its host.

### Parasitism and longevity

The higher number of eggs parasitized in the first 24 h, at 21 °C, 24 °C and 27 °C, may be due to the fact that *C. noackae* females are provigenic, that is, adults emerge with mature eggs ready to be laid (Jervis, Heimpel & Ferrns, 2001; Bari, Jahan & Islam, 2015). On the other hand, the lower number of eggs parasitized at 15 °C and 18 °C is due to the reduction in the metabolic rate of larvae of this parasitoid (Boivin, 2010) and may also be related to the lower probability of encountering, foraging and oviposition at lower temperatures (Gillooly et al., 2002; Hance et al., 2007; Abram et al., 2017). The high parasitism by *C. noackae* in the first 24 h at 21 °C and 27 °C is similar to that reported for the parasitoids *Trichogramma pretiosum* Riley (Hymenoptera: Trichogrammatidae) in eggs of *Pseudoplusia includens* Walker (Lepidoptera: Noctuidae) at 18 °C and 32 °C (Bueno, Parra & de Freitas Bueno, 2012) and *Telenomus remus* Nixon (Hymenoptera: Scelionidae) and *T. pretiosum* in eggs of *Spodoptera* spp. (Lepidoptera: Noctuidae) at 24 °C (Pinto & Fernandes, 2020). The short parasitism activity and longevity of this parasitoid (Mutitu et al., 2013; Souza et al., 2016) are important aspects for the success of biological control, as it reduces the period of exposure to biotic and abiotic factors, including pesticides and climate (Bueno, Parra & de Freitas Bueno, 2012; Laumann & Sampaio, 2020).

The greater total number of *T. peregrinus* eggs parasitized per *C. noackae* female, at 21 °C, 24 °C and 27 °C than at 30 °C can be explained by the restriction and reduction in the allocation of lipids, proteins and carbohydrates, at the latter temperature, during the larval stage (Visser & Ellers, 2008; Moiroux, Brodeur & Boivin, 2014; Abram et al., 2017). Moreover, parasitoids that develop at temperatures above or below the thermal tolerance limits are less productive, with fewer descendants (Colinet, Boivin & Hance, 2007). The reduction in the number of eggs parasitized by *C. noackae* at the highest temperature is similar to that reported for *A. inexpectatus* in eggs of *G. platensis*, 2.3 eggs at 30 °C (Valente et al., 2017). The increase in the temperature reduces the efficiency of foraging in hosts and, consequently, the longevity of parasitoids that have a finite lipid reserve and a finite number of eggs. Therefore, this natural enemy depends on finding hosts, within a short period, to oviposit and reach maximum fertility (Denis et al., 2011; Laumann & Sampaio, 2020).

The sex ratio of *C. noackae* from 0.57 to 0.69 between 15 °C and 30 °C and the higher number of females emerged at all temperatures are important factors for mass rearing parasitoids, because it is responsible for their rates of parasitism and their viability (Heimpel & Lundgren, 2000). This shows that temperature does not affect sex ratio of this parasitoid in mass rearing in the laboratory, which is important for the quality because the females are responsible for parasitism (Laumann & Sampaio, 2020). Additionally, temperature did not affect the spermatozoa retention behavior during oviposition (Charnov, 1982; Pereira et al., 2009), generating a lower number of males (Heimpel & Lundgren, 2000). The sex ratio, with a high number of *C. noackae* females of this parasitoid, at the different temperatures, is similar to that reported for other Mymaridae such as the parasitoid *G. ashmeadi* with 65% and 71% females at 15 °C and 33 °C (Pilkington & Hoddle, 2006) and *A. inexpectatus* with 54% and 62% females at 5 °C to 30 °C (Valente et al., 2017). The high percentage of *C. noackae* females at all temperatures differed from that reported for this parasitoid at 22 °C, 50% (Martínez, González & Dicke, 2018) and in zero to one-day-old *T. peregrinus* eggs, 78% to 24 °C (Barbosa et al., 2018). This indicates a high potential for population growth of this natural enemy, due to the greater number of females at all temperatures (Laumann & Sampaio, 2020).

The greater longevity of female and male *C. noackae* at the lower temperatures, except at 18 °C, may be associated to reduced activity and metabolism (Bleicher & Parra, 1989). On the other hand, the lower longevity of *C. noackae* at higher temperatures is similar to that reported for this parasitoid at 28 °C and 30 °C, 26.4 h (Souza et al., 2016) and for that of other Mymaridae, such as *A. nitens* and *A. inexpectatus* (Valente et al., 2017) and *G. ashmeadi* (Pilkington & Hoddle, 2006). This is due to an increase in the metabolic processes of ectotherm insects and a reduction in lipid reserves during embryonic development, with a negative effect on adult longevity (Huey & Stevenson, 1979; Colinet, Boivin & Hance, 2007; Keil, Cummings & de Magalhães, 2015) and on foraging for host eggs (Denis et al., 2011) and, consequently, leading to reduced efficiency in biological control.

Furthermore, the longevity of 50.76 h we observed at 24 °C compared to the 84 h at 25 °C for *C. noackae* females without parasitism experience (Souza et al., 2016) may indicate that this experience affects this parameter, possibly due to the energy expenditure during copulation and oviposition (Pacheco & Corrêa-Ferreira, 1998).

The greater *C. noackae* survival at 15 °C, 21 °C and 24 °C in the first 48 h than at 27 °C and 30 °C indicates that this parasitoid does not tolerate high temperatures that may be related to the increase in its metabolic processes and destruction of enzymes (Mohan, Verma & Singh, 1992) besides reduction in the nutritional reserves (Visser & Ellers, 2008; Moiroux, Brodeur & Boivin, 2014; Abram et al., 2017).

### Fertility life table of *Cleruchoides noackae*

The higher R_o_ of *C. noackae* at 21 °C and 24 °C than at 30 °C may be due to the higher temperature reducing the allocation of lipids, important for oogenesis (Ellers & van Alphen, 1997; Pexton & Mayhew, 2002) and for foraging and oviposition behavior (Denis et al., 2011). The net reproductive rate of the Mymaridae *G. ashmeadi* and *A. nitens* in *H. coagulata* and *G. platensis* eggs was also higher at 24 °C (Chen et al., 2006) and at 20 °C and 25 °C, respectively (Valente et al., 2017). Net reproductive rate >1 for *C. noackae* at all temperatures, except 30 °C indicates a population increase of this parasitoid (Post & Thompson, 2017).

The greater interval between generations (T) of *C. noackae* at 15 °C, 18 °C and 21 °C demonstrates a reduction in the parasitoid metabolism at these temperatures (Lactin et al., 1995; Briere et al., 1999; Bari, Jahan & Islam, 2015) and an increase in its development period (Damos & Savopoulou-Soultani, 2012; Laumann & Sampaio, 2020). The increase in the intervals between generations as the temperature decreased is similar to that of *C. noackae* in *T. peregrinus* eggs, 19 to 27 days from 22 °C to 18 °C in Uruguay (Martínez, González & Dicke, 2018), *A. atomus* in *E. decipiens* eggs, from 28 °C to 16 °C, 14.43 to 30.9 days (Agboka et al., 2004) and *A. inexpectatus* in eggs of *G. platensis*, 18.39 to 72.89 days from 25 °C to 10 °C (Valente et al., 2017).

The lower finite rate of population increase of *C. noackae* at 30 °C than at 21 °C, 24 °C and 27 °C is due to the higher temperature reducing the energy generated in the metabolic processes important for reproduction and population growth (Angilletta & Dunham, 2003; Colinet, Boivin & Hance, 2007). The finite rate of increase of the parasitoid *A. atomus* in *E. decipiens* eggs and *C. noackae* in *T. peregrinus* eggs was higher at 24 °C, with 1.21 (Agboka et al., 2004).

The higher intrinsic rate of population increase of *C. noackae* at 18 °C, 21 °C, 24 °C and 27 °C than at 30 °C indicates that the population of this parasitoid, is more successful and with a significant increase between 18 °C to 27 °C (Post & Thompson, 2017). A similar result was found for the *C. noakae* host, *T. peregrinus*, with a higher intrinsic rate of increase at 25 °C (0.046) but negative at 30 °C (Barbosa et al., 2019). The higher intrinsic rate of population increase of the parasitoid *C. noackae* than that of its host *T. peregrinus* between 18 °C and 30 °C (Barbosa et al., 2019) indicates the potential success of this natural enemy in the biological control of the target pest (Van Lenteren, 2009). The parasitoid intrinsic growth rate (rm), equal to or greater than that of its host (Barbosa et al., 2019), will increase is effectiveness in managing *T. peregrinus*. Evaluations at fixed and constant temperatures have limitations, because the variations of this parameter in the field affect parasitoid performance. The fact that this parasitoid is not suitable at higher temperatures, common in eucalyptus areas in Brazil, raises concern, and there is a need to develop strains tolerant to this condition. These results have important applications for the biological control of *T. peregrinus*, and the mass rearing of *C. noackae* and its potential for field establishment. The development of research with the effect of host and parasitoid density on parasitism efficiency will complement the integrated management of this pest.

## Conclusions

The temperatures of 21 °C and 24 °C and from 18 °C to 27 °C are the most suitable for the reproduction and population increase, respectively, of *C. noackae* in *T. peregrinus* eggs.

The best constant temperature to rear both *T. peregrinus* and *C. noackae* is 24 °C, to achieve higher production of parasitoids with preserved parasitism potential for mass release.

These results confirm mass-rearing protocols using similar conditions to optimize mass production of this parasitoid in the laboratory for biological control programs of *T. peregrinus* in eucalyptus plantations.

## Supplemental Information

10.7717/peerj.14911/supp-1 Supplemental Information 1 Data Longevity.

10.7717/peerj.14911/supp-2 Supplemental Information 2 Data parasitism.

10.7717/peerj.14911/supp-3 Supplemental Information 3 Data parasitism parental.

10.7717/peerj.14911/supp-4 Supplemental Information 4 Data survival—temperature.

## Additional Information and Declarations

### Data Availability

The following information was supplied regarding data availability: The raw measurements are available in the Supplemental Files.
